# Perioperative Outcomes of Video-Assisted Thoracoscopic Surgery Versus Open Thoracotomy After Neoadjuvant Chemoimmunotherapy in Resectable NSCLC

**DOI:** 10.3389/fonc.2022.858189

**Published:** 2022-05-31

**Authors:** Baihua Zhang, Qin Xiao, Haifan Xiao, Jie Wu, Desong Yang, Jinming Tang, Xu Li, Zhining Wu, Yong Zhou, Wenxiang Wang

**Affiliations:** ^1^ The Second Department of Thoracic Surgery, Hunan Clinical Medical Research Center of Accurate Diagnosis and Treatment for Esophageal Carcinoma, Hunan Cancer Hospital and The Affiliated Cancer Hospital of Xiangya School of Medicine, Central South University, Changsha, China; ^2^ Key Laboratory of Translational Radiation Oncology, The First Department of Thoracic Radiation Oncology, Hunan Cancer Hospital, The Affiliated Cancer Hospital of Xiangya School of Medicine, Central South University, Changsha, China; ^3^ Cancer Prevention Office, Hunan Cancer Hospital and The Affiliated Cancer Hospital of Xiangya School of Medicine, Central South University, Changsha, China

**Keywords:** non-small cell lung cancer (NSCLC), neoadjuvant therapy, immunotherapy, video assisted thoracoscopic surgery (VATS), thoracotomy

## Abstract

**Background:**

Neoadjuvant chemoimmunotherapy becomes more widespread in the treatment of NSCLC, but few studies have reported the details of surgical techniques and perioperative challenges following neoadjuvant chemoimmunotherapy until now. The primary aim of our study was to address the feasibility and safety of pulmonary resection after neoadjuvant chemoimmunotherapy *via* different surgical approaches, video-assisted thoracoscopic surgery (VATS) and open thoracotomy.

**Methods:**

Patients with an initial diagnosis of clinical stage IB-IIIB(T3-4N2) NSCLC, who received neoadjuvant chemoimmunotherapy and surgery between January 2019 and August 2021 were included. Patients were retrospectively divided into two groups (VATS, and thoracotomy), and differences in perioperative, oncological, and survival outcomes were compared.

**Results:**

In total, there were 131 NSCLC patients included. Surgery was delayed beyond 42 days in 21 patients (16.0%), and radical resection (R0) was achieved in 125 cases (95.4%). Lobectomy was the principal method of pulmonary resection (102 cases, 77.9%) and pneumonectomy was performed in 11 cases (8.4%). Postoperative complications within 30 days occurred in 28 patients (21.4%), and no 90-day mortality was recorded. There were 53 patients (38.5%) treated with VATS, and 78 (59.5%) with open thoracotomy. VATS could achieve similar definitive resection rates, postoperative recovery courses, comparable morbidities, and equivalent RFS rates(p>0.05), with the advantages of reduced operative time (160.1 ± 40.4 *vs* 177.7 ± 57.7 min, p=0.042), less intraoperative blood loss (149.8 ± 57.9 *vs* 321.2 ± 72.3 ml, p=0.021), and fewer intensive care unit(ICU) stays after surgery (3.8% *vs* 20.5%, p=0.006) compared with open thoracotomy. However, the mean number of total lymph nodes resected was lower in the VATS group (19.5 ± 7.9 *vs* 23.0 ± 8.1, p=0.013). More patients in the thoracotomy group received bronchial sleeve resection/bronchoplasty (53.8% *vs* 32.1%, p=0.014) and vascular sleeve resection/angioplasty (23.1% *vs* 3.8%, p=0.003). After propensity score matching (PSM) analysis, VATS still had the advantage of fewer ICU stays after surgery (2.3% *vs*. 20.5%, p=0.007).

**Conclusions:**

Our results have confirmed that pulmonary resection following neoadjuvant PD-1 inhibitors plus chemotherapy is safe and feasible. VATS could achieve similar safety, definitive surgical resection, postoperative recovery, and equivalent oncological efficacy as open thoracotomy, with the advantage of fewer ICU stays after surgery.

## Introduction

Surgery following neoadjuvant therapy has been proposed as one of the principal multidisciplinary treatments to achieve improved local and distant disease control in resectable non-small cell lung cancer (NSCLC). Emerging data from various ongoing phase II studies have indicated that the addition of programmed cell death protein 1 (PD-1)/programmed cell death ligand 1(PD-L1) inhibitors alone or in combination with chemotherapy before surgery may significantly improve the survival of resectable NSCLC (1–5). Recently, the checkmate 816 trial, which was the only published phase III study on neoadjuvant chemoimmunotherapy until now, has shown a 24.0% pathological complete response (pCR) rate in patients treated with neoadjuvant Nivolumab combined with platinum doublets, approximately 10-fold higher than in patients receiving chemotherapy alone (2.2%) (6). These clinical trials have established the positive role of PD-1 inhibitors plus chemotherapy in the neoadjuvant treatment of NSCLC.

Surgical resection is still considered as the ultimate curative treatment for NSCLC when complete resection is feasible (7). However, as neoadjuvant chemoimmunotherapy becomes more widespread in clinical practice, there are increasing debates about the use of surgery. Considering that the hilar and mediastinal structure might be affected by neoadjuvant PD-1/PD-L1 inhibitors and chemotherapy, the preferable surgical approaches and surgical techniques require further clarification. But few studies have reported the details of surgical techniques and perioperative challenges following neoadjuvant chemoimmunotherapy until now.

Furthermore, video-assisted thoracic surgery (VATS) is superior to open thoracotomy for the resection of early stage NSCLC, with the advantages of less complications, less pain, faster recovery and equivalent long-term survival (8–11); however, several recent case series and anecdotal reports have suggested that VATS following neoadjuvant chemotherapy could also achieve satisfactory outcomes in locally advanced NSCLC patients (2, 12–14). But there was little evidence suggesting the feasibility and safety of VATS after neoadjuvant chemoimmunotherapy in NSCLC. It is widely acknowledged that neoadjuvant therapy might result in dense adhesions and fibrosis in the chest (1, 15, 16), particularly in cases with significant treatment response, which might increase the complexity of the surgical field. As down-staging rates are significantly increased after neoadjuvant PD-1 inhibitors plus chemotherapy, whether VATS remains suitable for patients with smaller down-staged tumors, and whether there will be increased rates of complications and conversion to thoracotomy remain to be clarified. Moreover, a comparative analysis between VATS and open thoracotomy following neoadjuvant PD-1 inhibitors plus chemotherapy in NSCLC has not yet been performed.

This study was conducted to investigate the perioperative outcomes of surgery following neoadjuvant PD-1 inhibitors plus chemotherapy in resectable NSCLC. The primary aim was to address the feasibility and safety of pulmonary resection after neoadjuvant chemoimmunotherapy *via* different surgical approaches, VATS and open thoracotomy. The advantages and disadvantages of VATS following neoadjuvant PD-1 inhibitors plus chemotherapy was also identified compared with open thoracotomy.

## Patients and Methods

### Patient Selection

This study was a retrospective, single-center, real-world observational study. The study protocol was approved by the Ethics Committee of Hunan Cancer Hospital and informed consent was waived due to the retrospective nature of the study (Approval No. 2021-44, Aug 31^st^, 2021). The study was conducted in accordance with the Declaration of Helsinki (as revised in 2013). Patients with an initial diagnosis of clinical stage IB–IIIB (American Joint Committee on Cancer, 8^th^ edition) resectable NSCLC, who received neoadjuvant PD-1 inhibitors plus chemotherapy prior to surgery from January 2019 to August 2021 at Hunan Cancer Hospital were included. Patients with stage IIIB NSCLC only included those in stage T3-4N2M0 (single N2 station), while those with N3 diseases were excluded in the present study. All patients had an Eastern Cooperative Oncology Group performance-status score of 0 or 1. Patients who failed to undergo surgical resection due to any reasons were excluded. All clinical data, including demographic characteristics, clinicopathological information, and postoperative morbidity and mortality were obtained from medical records, and a retrospective analysis was performed. The clinical stages were reviewed by three senior doctors including a radiologist, physician, and surgeon. All staging decisions were based on the AJCC 8th staging system, and clinical staging was confirmed if at least two doctors agreed. Primary tumor length before treatment was defined as the maximum diameter assessed by computed tomography (CT) or positron-emission tomography/CT scans one month before initial treatment.

### Neoadjuvant Treatment

This study included 131 patients treated with neoadjuvant PD-1 inhibitors plus chemotherapy. All patients received one to five cycles of conventional platinum-based doublet chemotherapy (21 d per cycle), with PD-1 inhibitors given on day 1 of each chemotherapy cycle. The PD-1 inhibitors included Toripalimab, Pembrolizumab, Sintilizumab, Nivolizumab, Camrelizumab, and Tislelizumab. Pathological treatment response was graded, with major pathological response (MPR) defined as <10% residual viable tumor cells (17). Radiographic evaluations including PET-CT, or/and chest and abdominal CT, brain magnetic resonance imaging (MRI), bone SPECT, were performed before surgery.

### Surgical Treatment

After the discussion of a multidisciplinary team (MDT), curative-intent resection would be carried out after the recovery of neoadjuvant treatment. Based on the surgical approaches, patients were retrospectively divided into two groups: VATS, patients who received a two or three ports thoracoscopic approach; and open thoracotomy, patients who received conventional posterolateral serratus divided thoracotomies. Patients who underwent conversion from VATS to open thoracotomy were also included into open thoracotomy group. Surgery with curative-intent was performed in all patients, and radical (R0) resection was defined as microscopically negative margins, resection of at least three N1 and three N2 lymph node stations including station 7, and no extracapsular extension in resected lymph nodes (18). The chest tube was removed when no air leak was clearly confirmed, and the volume of drainage was <200 mL/d.

### Statistical Analysis

Continuous and categorical variables were analyzed using the Student’s t-test with distribution normality, and χ2 test (or Mann–Whitney U test and Fisher’s exact test), respectively. Distribution normality was tested by Kolmogorov-Smirov test. Generally, surgeons would choose the surgical approaches mainly based on the tumor features, including tumor length, location, T, N, TNM stage, and even radiographic tumor response after treatment. To balance such baseline characteristics between the groups, propensity score matching (PSM) with a ratio of 1:1 was performed using the nearest-neighbor method, without replacement and 0.02-caliper width. Recurrence-free survival (RFS) was defined as the length of time (in months) between the date of surgery and the date of confirmed disease progression or death from any cause. Survival analyses were calculated and compared using Kaplan–Meier curves and the log-rank test. SPSS version 23.0 software (IBM Corp., Armonk, NY, USA) was used to perform all statistical analyses. A P-value <0.05 (two-sided) was considered statistically significant.

## Results

### Baseline Characteristics of the Complete Cohort

In total, 131 patients with resectable NSCLC received neoadjuvant PD-1 inhibitor plus chemotherapy and included into further analysis. As summarized in **Table 1**, the mean age of the complete cohort was 59.3 ± 7.2 years. Men comprised 96.9% of the cohort. There was cigarette consumption in 120 patients (91.6%). The principal histological types were squamous cell carcinoma (105 cases, 80.2%), and adenocarcinoma (23 cases, 17.6%). The average tumor length before therapy was 5.0 ± 1.9 cm. There were 101 cases with central tumors (77.1%). Clinical staging before treatment was as follows: 31 (23.7%) stage IB-IIB (T2-3N0, T1-2N1), 66 (50.4%) stage IIIA (T1-2N2, T3N1, T4N0-1), and 34 (26.0%) stage IIIB [T3-4N2M0(single N2 station)].

**Table 1:** Baseline characteristics for 131 NSCLC patients received neoadjuvant PD-1 inhibitors plus chemotherapy.

Characteristics		Value (%)
Age	`x ± s^a,^ y	59.3 ± 7.2
Gender	Male	127 (96.9)
	Female	4 (3.1)
Smoking habits	Non-smoker	11 (8.4)
	Present/ex-smoker	120 (91.6)
Pathological type	Squamous cell carcinoma	105 (80.2)
	Adenocarcinoma	23 (17.6)
	Adenosquamous cell carcinoma	2 (1.5)
	Sarcomatoid carcinoma	1 (0.8)
Tumor length before therapy	`x ± s^a,^ cm	5.0 ± 1.9
Tumor location	Peripheral	30 (22.9)
	Central	101 (77.1)
cT stage	T1-3	100 (76.3)
	T4	31 (23.7)
cN stage	N0	15 (11.5)
	N1	48 (36.6)
	N2	68 (51.9)
cTNM stage	IB- IIB	31 (23.7)
	IIIA	66 (50.4)
	IIIB	34 (26.0)
PD-1 inhibitors	Pembrolizumab	34 (26.0)
	Sintilizumab	26 (19.8)
	Toripalimab	24 (18.3)
	Camrelizumab	20 (15.3)
	Nivolizumab	17 (13.0)
	Tislelizumab	10 (7.6)
Radiographic tumor response	PR	103 (78.6)
	SD/PD	28 (21.4)
Neoadjuvant therapy cycles	< 3	88 (67.2)
	≥ 3	43 (32.8)
Interval time between final neoadjuvant therapy and surgery(day)	≤ 42d	110 (84.0)
	> 42d	21 (16.0)
Surgical approach	VATS	53 (40.5%)
	Thoracotomy	78 (59.5%)
Surgical radicality	Radical	125 (95.4)
	Palliative	6 (4.6)
Extent of resection	Lobectomy	102 (77.9)
	Bilobectomy	18 (13.7)
	Pneumonectomy	11 (8.4)
Bronchial sleeve resection/bronchoplasty	Yes	59 (45.0)
	No	72 (55.0)
Vascular sleeve resection/angioplasty	Yes	20 (15.3)
	No	111 (84.7)
Postoperative complications	Yes	28 (21.4)
	No	103 (78.6)
Pathological response	MPR	70 (53.4)
	PR	45 (34.4)
	SD+PD	16 (12.2)

a Variables were described by mean (x) and standard deviation(s). NSCLC, non-small cell lung cancer; cT, clinical T stage before treatment; cN, clinical N stage before treatment; cTNM, clinical TNM stage before treatment, stage IB-IIB include T2-3N0, T1-2N1, stage IIIA include T1-2N2, T3N1, T4N0-1, and stage IIIB include T3-4N2M0(single N2 station); VATS, video-assisted thoracic surgery; mPR, major pathological response; PR, partial response; SD, stable disease; PD, progressive disease.

All patients received one to five cycles of neoadjuvant therapy before surgery, and 43 patients (32.8%) received ≥3 cycles of neoadjuvant therapy. Radiographic tumor regression (PR) was detected in 103 patients (78.6%) before surgery. The median interval time between neoadjuvant therapy termination and surgery was 32 days (range: 19–90 days). Surgery was delayed beyond 42 days after the final neoadjuvant therapy in 21 patients (16.0%) due to treatment-related adverse events (16 patients) and economic reasons or hesitation to undergo surgery (5 patients). Resection with curative-intent including removal of at least three N1 and three N2 lymph node stations including station 7 was performed in all patients. Radical resection (R0) was achieved in 125 cases (95.4%), and another six patients received palliative resection (R1) or uncertain resection, including microscopically positive margins in three patients and positive highest lymph node in three patients.

There were 53 patients (40.5%) received pulmonary resection *via* VATS, and other 78 patients *via* conversion/open thoracotomy. Lobectomy was the principal method of pulmonary resection (102 cases, 77.9%) in the present study, and pneumonectomy was performed in 8.4% of the cohort. There were 59 patients (45.0%) received bronchial sleeve resection/bronchoplasty, while vascular sleeve/angioplasty was performed in 20 patients (15.3%). Postoperative complications within 30 days occurred in 28 patients (21.4%). The principal types of complications are summarized in detail in **Table 2**. Postoperative pneumonia, prolonged air leak, and arrhythmia/heart failure were the most frequent complications. Only two patients had immunological hepatitis after surgery, and no 90-day mortality was recorded.

**Table 2:** Postoperative complications within 30 days and 90-day morbidity in different surgical approaches.

Complications	VATS(n=10) (%)	Thoracotomy (n=18) (%)	P
Pneumonia	2 (20.0)	8 (44.4)	0.247
Prolonged Air leak	5 (50.0)	1 (5.6)	0.013
Respiratory failure	0	2 (11.1)	0.524
Arrhythmia/heart failure	1 (10.0)	3 (16.7)	1.000
Chylothorax	0	1 (5.6)	1.000
Hoarseness	1 (10.0)	0	1.000
Immunologic hepatitis	0	2 (11.1)	0.524
Urinary retention	1 (10.0)	0	1.000
Vomiting/nausea	0	1 (5.6)	1.000
90-day mortality	0	0	1.000

### Comparison Between VATS and Open Thoracotomy

In total, 53 patients (40.5%) were treated with VATS, and 78 (59.5%) with open thoracotomy, including 42 patients with conversion surgery. As summarized in **Table 3**, there were relatively more patients with smaller tumors in the VATS group than in the thoracotomy group (average length, 4.6 ± 1.7 vs 5.2 ± 2.0 cm, p=0.040). More patients with stage T1–3 disease seemed to receive VATS (88.7% *vs* 67.9%, p=0.006). The percentage of earlier-staged tumors (cIB–IIB) was up to 34.0% in the VATS group, which was higher than in the other group (p=0.008). Meanwhile, tumor location, cN stage and radiographic tumor response were comparable between VATS and open thoracotomy.

**Table 3:** Comparison of tumor characteristics for 131 NSCLC patients received pulmonary resection *via* VATS or thoracotomy.

Variable		Before PSM					After PSM		
		VATS (n = 53)	Thoracotomy (n = 78)	P value	SMDb	VATS (n = 44)	Thoracotomy (n = 44)	P value	SMDb
Age	$\bar{\text{x}}\pm s^{\text{a}}$	59.6 ± 6.21	59.1 ± 7.8	0.696	0.071	59.4 ± 6.0	59.6 ± 7.7	0.853	0.04
Gender	Male	52 (98.1)	75 (96.2)	0.522	0.117	43 (97.7)	43 (97.7)	1.000	<0.001
	Female	1 (1.9)	3 (3.8)			1 (2.3)	1 (2.3)		
Smoking habits	Non-smoker	3 (5.7)	8 (10.3)	0.352	0.169	2 (4.5)	6 (13.6)	0.138	0.317
	Present/ex-smoker	50 (94.3)	70 (89.7)			42 (95.5)	38 (86.4)		
Pathological type	Squamous cell carcinoma	38 (71.7)	68 (87.2)	0.027	0.387	35 (79.5)	38 (86.4)	0.395	0.180
	Non-Squamous cell carcinoma	15(28.3)	10(12.8)			9(20.5)	6(13.6)		
Tumor length before therapy	$\bar{\text{x}}\pm s^{\text{a}}$	4.6 ± 1.7	5.2 ± 2.0	0.040	0.364	4.6 ± 1.8	4.7 ± 1.9	0.890	0.030
Tumor location	Peripheral	15 (28.3)	15 (19.2)	0.225	0.213	10 (22.7)	10 (22.7)	1.00	<0.001
	Central	38 (71.7)	63 (80.8)			34 (77.3)	34 (77.3)		
cT stage	T1-3	47 (88.7)	53 (67.9)	0.006	0.516	38 (86.4)	39 (88.6)	0.747	0.068
	T4	6 (11.3)	25 (32.1)			6 (13.6)	5 (11.4)		
cN stage	N0	7 (13.2)	8 (10.3)	0.142	0.298	5 (11.4)	4 (9.1)	0.933	0.034
	N1	24 (45.3)	24 (30.8)			17 (38.6)	18 (40.9)		
	N2	22 (41.5)	46 (59.0)			22 (50.0)	22 (50.0)		
cTNM stage	IB- IIB	18 (34.0)	13 (16.7)	0.008	0.570	11 (25.0)	12 (27.3)	0.788	0.034
	IIIA	28 (52.8)	38 (48.7)			26 (59.1)	23 (52.3)		
	IIIB	7 (13.2)	27 (34.6)			7 (15.9)	9 (20.5)		
Radiographic tumor regression	PR	41 (77.4)	62 (79.5)	0.771	0.051	36 (81.8)	34 (77.3)	0.597	0.112
	SD/PD	12 (22.6)	16 (20.5)			8 (18.2)	10 (22.7)		
Surgical radicality	Radical	51 (96.2)	74 (94.9)	0.716	0.106	42 (95.5)	41 (93.2)	0.645	0.097
	Palliative/Exploration	2 (3.8)	4 (5.1)			2 (4.5)	3 (6.8)		
Operative time	x ± s^a^(min)	160.1 ± 40.4	177.7 ± 57.7	0.042	0.353	164.5 ± 38.8	169.2 ± 59.5	0.659	0.095
Intraoperative blood loss	x ± s^a^(ml)	149.8 ± 57.9	321.2 ± 638.8	0.021	0.378	147.5 ± 59.8	346.6 ± 823.6	0.113	0.341
Extent of resection	Lobectomy	45 (84.9)	57 (73.1)	0.201	0.270	37(84.1)	32(72.7)	0.208	0.168
	Bilobectomy	6 (11.3)	12 (15.4)			2(4.5)	7(15.9)		
	Pneumonectomy	2 (3.8)	9 (11.5)			5(11.4)	5(11.4)		
Bronchial sleeve resection/bronchoplasty	Yes	17 (32.1)	42 (53.8)	0.014	0.447	14(31.8)	22(50.0)	0.083	0.372
	No	36 (67.9)	36 (46.2)			30(68.2)	22(50.0)		
Vascular sleeve resection/angioplasty	Yes	2 (3.8)	18 (23.1)	0.003	0.586	1(2.3)	8(18.2)	0.014	0.538
	No	51 (96.2)	60 (76.9)			43(97.7)	36(81.8)		
Number of total lymph nodes	x ± s^a^	19.5 ± 7.9	23.0 ± 8.1	0.013	0.447	19.0 ± 5.3	22.2 ± 7.3	0.021	0.503
Number of resected N1 nodes	x ± s^a^	7.4 ± 4.3	8.5 ± 3.9	0.116	0.285	6.9 ± 2.9	8.2 ± 3.6	0.061	0.405
Number of resected N2 nodes	x ± s^a^	12.1 ± 5.0	14.3 ± 6.5	0.031	0.379	12.1 ± 4.2	13.9 ± 5.6	0.095	0.360
Total drainage after operation	x ± s^a^(ml)	1123.6 ± 531.9	1307.2 ± 662.4	0.082	0.306	1149.0 ± 516.7	1201.7 ± 568.6	0.650	0.097
Duration of chest tube	x ± s^a^(day)	5.5 ± 4.6	5.3 ± 2.2	0.830	0.041	5.7 ± 4.9	4.9 ± 1.8	0.343	0.203
ICU stay after surgery	Yes	2 (3.8)	16 (20.5)	0.006	0.526	1(2.3)	9(20.5)	0.007	0.591
	No	51 (96.2)	62 (79.5)			43(97.7)	35(79.5)		
Hospital stay after surgery	x ± s ^a^(day)	6.7 ± 3.9	7.2 ± 3.2	0.386	0.158	6.8 ± 4.2	6.9 ± 3.0	0.907	0.025
Pathological response	mPR	30 (56.6)	40 (51.3)	0.693	0.144	27(61.4)	20(45.5)	0.280	0.340
	PR	18 (34.0)	27 (34.6)			14(31.8)	18(40.9)		
	SD+PD	5 (9.4)	11 (14.1)			3(6.8)	6(13.6)		

a Variables were described by mean (**x**) and standard deviation(**s**). Variable means standard mean difference, imbalance between treatment groups was defined as an SMD ≥ 0.1; balance between treatment groups was defined as an SMD <0.1. NSCLC, non-small cell lung cancer; PD-1+C, PD-1 inhibitor plus chemotherapy; Chemo, chemotherapy; cT, clinical T stage before treatment; cN, clinical N stage before treatment; cTNM, clinical TNM stage before treatment, stage IB-IIB include T2-3N0, T1-2N1, stage IIIA include T1-2N2, T3N1, T4N0-1, and stage IIIB include T3-4N2M0(single N2 station); VATS, video-assisted thoracic surgery; ICU, intensive care unit.

The mean operative time in the VATS group was 160.1 ± 40.4 min, shorter than that of 177.7 ± 57.7 min in the thoracotomy group(p=0.042). The average intraoperative blood loss was 149.8 ± 57.9 ml in the VATS group, which was decreased compared with the other group (321.2 ± 72.3 ml, p=0.021), indicating less operative time and blood loss during surgery was one of the advantages of VATS. However, no significant differences were detected in surgical radicality and extent of resection between groups. However, the frequency of bronchial sleeve resection/bronchoplasty was 32.1% in the VATS group, which was less than the 53.8% in the thoracotomy group (p=0.014). Vascular sleeve resection/angioplasty was performed in only 3.8% of patients in the VATS group, which was significantly lower than the 23.1% in the thoracotomy group (p=0.003). The mean number of total lymph nodes resected in the VATS group was 19.5 ± 7.9, which was lower than in the thoracotomy group (23.0 ± 8.1, p=0.013). Further analysis identified that less N2 lymph nodes were resected in the VATS group (12.1 ± 5.0 *vs* 14.3 ± 6.5, p=0.031), while similar N1 lymph nodes were harvested in both groups.

After surgery, only 2 patients in the VATS groups required ICU stay, less than 16 patients in the thoracotomy group (p=0.006). Regarding other postoperative parameters including total drainage after operation, duration of chest tube, hospital stay after surgery, and pathological response, no significant differences were detected between groups.

After six baseline characteristics including tumor length before therapy, tumor location, cT stage, cN stage, cTNM stage, and radiographic tumor response were balanced between the VATS and thoracotomy groups, 44 patients from each group were further incorporated into PSM analysis (**Table 3**). The surgical data showed that VATS had the advantages of fewer ICU stays after surgery (2.3% *vs*. 20.5%, p=0.007), while no significant difference was detected in operative time, intraoperative blood loss, and bronchial sleeve resection/bronchoplasty between groups. Vascular sleeve resection/angioplasty (2.3% *vs*. 18.2%, p=0.014) were more likely to be performed *via* open thoracotomy. However, the mean number of lymph nodes resected in the VATS group (19.0 ± 5.3) was lower than that in the thoracotomy group (22.2 ± 7.3, p=0.021). Finally, no significant differences were observed between other intraoperative and postoperative factors.

### Reasons for Unexpected Conversion From VATS to Open Thoracotomy

There were 95 patients scheduled to receive VATS; however, conversion from VATS to open thoracotomy was performed in 42, making the conversion rate 44.2%. Among them (**Table 4**), the principal causes for conversion surgery were primary tumor invasion (19 cases, 45.2%), dense adhesion and fibrosis after treatment (11 cases, 26.2%), fibrocalcified lymph nodes (6 cases, 14.3%), and pleural adhesion (2 cases, 6.9%). However, another four patients received unexpected conversion to open thoracotomy due to intraoperative vascular injury and hemorrhage.

**Table 4:** Reasons for unexpected conversion from VATS to open thoracotomy.

Reasons	N=42 (100%)
Primary tumor invasion	19 (45.2)
Dense adhesion and fibrosis after treatment	11 (26.2)
Fibrocalcified lymph nodes	6 (14.3)
Pleural adhesion	2 (4.8)
Intraoperative bleeding	4 (9.5)

As summarized in **Table 3**, compared with the VATS group, more patients with clinical stage IIIB(T3-4N2) NSCLC received thoracotomy (13.2% *vs*. 34.6%, p=0.008), and the frequency of vascular sleeve resection/angioplasty and bronchial sleeve resection/bronchoplasty was also higher in the thoracotomy group, indicating that advanced cTNM stage before treatment and more difficulty in pulmonary resection might be related with open thoracotomy.

### Anti-Tumor Pathological Responses and Survival Outcomes

Among the whole cohort, postoperative pathological results showed MPR in 70 patients (53.4%). No correlation between surgical approaches and pathological response was observed in this study.

**Figure 1:**
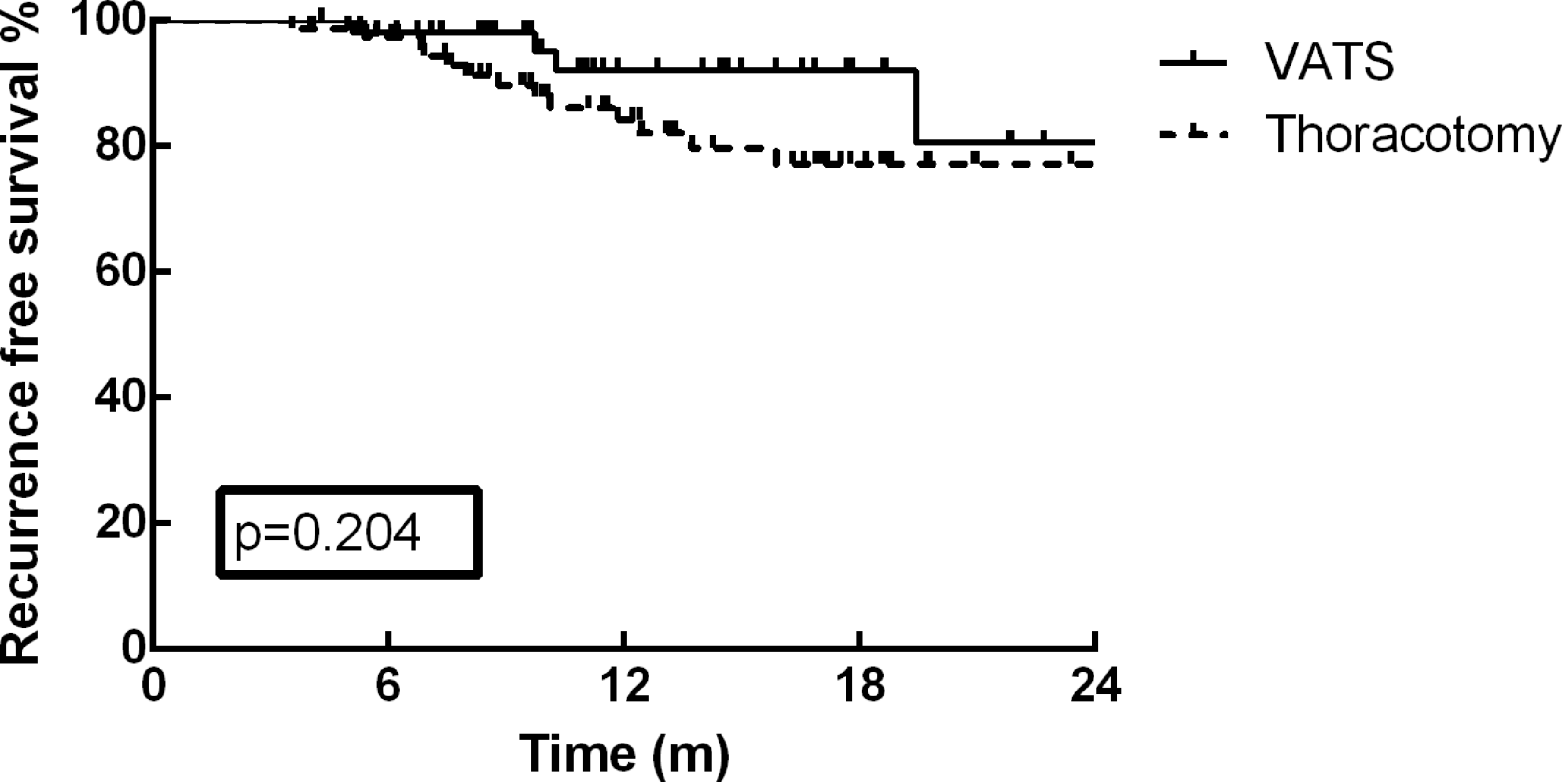
No survival difference was identified between the different surgical approaches (p=0.204).

**Figure 2:**
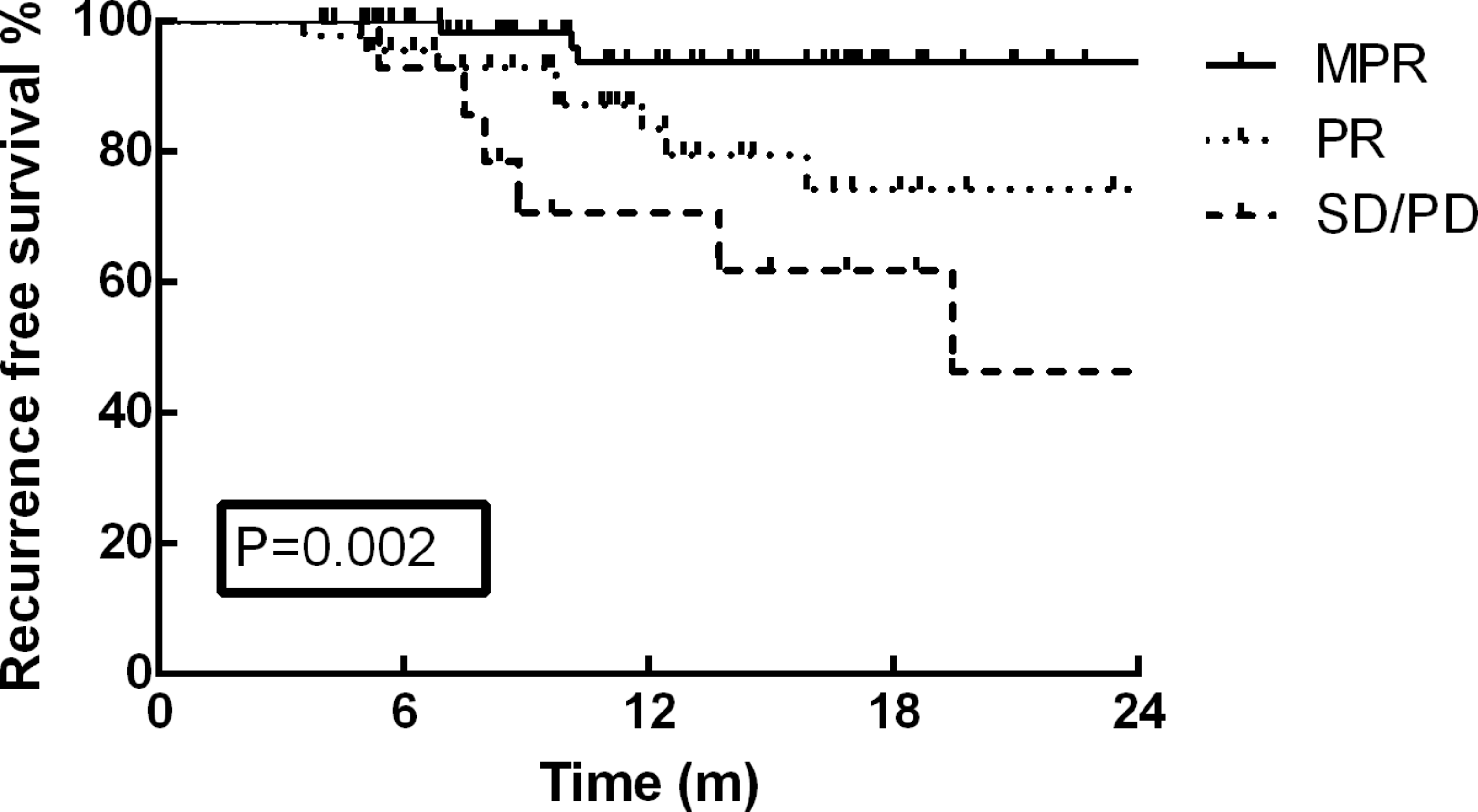
The RFS rate of patients in MPR group was much better than that of PR and SD/PD groups in the present study (p=0.002).

As of December 30, 2021, the median follow-up time was 13.2 months (range: 3.7–35.9 months). At the end of the current follow-up, tumor recurrence occurred in 17 patients (13.0%). The first failure sites among these 17 patients included distant metastasis in 12 patients, local recurrence in 4 patients, and concurrent distant and local metastasis in one patient. The most frequent distant metastasis occurred in brain (5 cases), and pulmonary (3 cases). On the basis of these results, no significant difference was detected in tumor recurrence between VATS and thoracotomy (p=0.127). Furthermore, the one-year RFS rate of the cohort was 87.2%, and no survival difference was identified between the different surgical approaches (**Figure 1**, p=0.204). The RFS rate of patients in MPR group was much better than that of PR and SD/PD groups in the present study (**Figure 2**, p=0.002).

## Discussion

As the use of neoadjuvant chemoimmunotherapy is increasing rapidly in locally advanced NSCLC, it is incumbent on surgeons to cope with the new perioperative challenges under this novel treatment strategy. This study represents one of the largest experiences to date of neoadjuvant chemoimmunotherapy in resectable NSCLC from real-world observations. Our study has shown that neoadjuvant chemoimmunotherapy did not increase perioperative challenges. Only 21 patients (16.0%) received delayed surgery beyond 42 days after the final cycle of neoadjuvant therapy, and radical resection (R0) was achieved in 95.4% of the cohort. As previously reported (2, 5), lobectomy was the principal method of pulmonary resection (102 cases, 77.9%), and pneumonectomy was performed in 8.4% of the cohort (11 cases). There were 59 patients (45.0%) received bronchial sleeve resection/bronchoplasty, while vascular sleeve/angioplasty was performed in 20 patients (15.3%) in the present study. The MPR rate of the cohort was 53.4%, in accordance with several previous prospective studies (1–6).

In this study, postoperative complications within 30 days occurred in 28 patients (21.4%). The principal types of postoperative complications included pneumonia, prolonged air leak, and arrhythmia/heart failure, which were in accordance with previous reports (2, 5, 8, 15). Only two patients had immunological hepatitis within 30 days after surgery, and no mortality occurred in this study, proving that the addition of PD-1 inhibitors before surgery did not increase severe postoperative complications, in accordance with previous results (2, 5).

In the phase III checkmate 816 study (6), 30% and 22% of patients received VATS in the Nivolumab + chemotherapy and chemotherapy-only groups, respectively. In a systemic review by Cao et al (2), between 26% and 46% of patients received VATS after neoadjuvant immunotherapy, which was identical to the 40.5% of patients in our study, suggesting that VATS remains one of the principal surgical approaches for NSCLC patients after neoadjuvant chemoimmunotherapy. Our surgical data have shown that VATS tended to be performed in patients with smaller size and early-stage (cT1-3, cIB–IIB) NSCLC. VATS following neoadjuvant PD-1 inhibitors plus chemotherapy could also achieve similar definitive resection rates, postoperative recovery courses, and comparable morbidities, with the advantages of reduced operative time, less intraoperative blood loss, and fewer ICU stays after surgery compared with open thoracotomy, which was also in accordance with previous studies (5, 12, 19). However, there may be biases in the comparison because more patients in the thoracotomy group received bronchial sleeve resection/bronchoplasty and/or vascular sleeve resection/angioplasty. Therefore, a PSM analysis in order to balance the tumor stages, location and other characteristics between groups was performed, and VATS still has the advantage of fewer ICU stays after surgery in the present study.

However, the average number of total and N2 lymph nodes resected in the VATS group was both lower than in the open thoracotomy group. Retrospective multicenter studies (11, 20) have reported that although there is a reduced average number of resected lymph nodes during VATS, long-term survival is still better than open thoracotomy. Zhang et al (21). and Tian et al (22). reported that similar numbers of total and N2 lymph node stations could be harvested *via* VATS following neoadjuvant therapy. Fortunately, at least three N1 and three N2 lymph node stations including station 7 were resected in each patient in our study. Several studies have reported that VATS following neoadjuvant chemotherapy could achieve similar long-term survival results as open thoracotomy in locally advanced NSCLC patients (2, 12, 16, 19). In accordance, no significant difference was observed in tumor recurrence or RFS rates between VATS and open thoracotomy in this study.

One of the controversies that surrounds using VATS following neoadjuvant therapy is whether the rates of conversion from the minimally invasive approach to open thoracotomy and perioperative morbidities would be increased. According to analyses of the National Cancer Database by Krantz et al. (23) and Yang et al. (12), pulmonary resection was ultimately accomplished *via* a conversion approach in 5.3% to 25.7% of locally advanced NSCLC patients after neoadjuvant chemotherapy with comparable morbidity and mortality. In other previous reports (1, 5, 24), conversion surgery is required in 25% to 54% of patients initially operated on *via* a VATS or robotic approach after neoadjuvant immunotherapy, while that rate was 42% in this study. Our results identified that primary tumor invasion and dense adhesion and fibrosis after treatment were the principal causes leading to conversion surgery after neoadjuvant chemoimmunotherapy, similar to what was reported by Bott et al (1). Several previous reports have suggested that neoadjuvant PD-1/PD-L1 inhibitors generate significant adhesion and fibrosis or “pseudo-progression” in the chest that can increase surgical complexity (1–3, 5). The extent of this effect is heterogeneous and unpredictable from restaging imaging prior to surgery (2, 3). Unfortunately, no correlation between surgical approaches and radiographic tumor regression or pathological response was identified in this study, either. Moreover, most of bronchial sleeve resection/bronchoplasty and vascular sleeve resection/angioplasty were performed *via* thoracotomy in our study, indicating that more complicated pulmonary resections might also increase the conversion rate.

The frequency and severity of principal postoperative complications in the VATS group was also comparable with the open thoracotomy. Regarding postoperative recovery courses, including total drainage after operation, duration of chest tube, hospital stay after surgery, there were no significant differences between VATS and open thoracotomy, which is in accordance with the surgical results after neoadjuvant chemotherapy (5, 9, 19). Therefore, our results suggest a positive role of VATS in the surgical treatment of NSCLC after neoadjuvant chemoimmunotherapy with equivalent safety to thoracotomy.

There were several limitations to this study. First, due to the retrospective analysis, potential bias was inevitable. Second, because only small cohorts of retrospective data were available, full assessments of the perioperative risk and surgical approaches after neoadjuvant chemoimmunotherapy will require further surgical information from the ongoing phase III studies. Third, because of the short follow-up time, long-term survival and recurrence information are lacking. Moreover, the choice of surgical approaches is personalized, according to cancer characteristics, surgeons’ experience, and preference, et al. Candidates for VATS among NSCLC patients treated with neoadjuvant chemoimmunotherapy require careful selection by experienced surgeons, and the decision to convert to open thoracotomy should be made promptly to avoid possible morbidity.

In conclusion, the present study reported the details of surgical techniques and perioperative challenges following neoadjuvant chemoimmunotherapy. Our results confirmed that pulmonary resection following neoadjuvant PD-1 inhibitors plus chemotherapy was safe and feasible. VATS could achieve similar safety, definitive surgical resection, postoperative recovery, and equivalent oncological efficacy as open thoracotomy, with the advantages of fewer ICU stays after surgery. Therefore, both VATS and open thoracotomy should be recommended as principal surgical approaches for curative-intent resection in selected NSCLC patients. However, appropriate candidates to receive VATS should be carefully screened before surgery.

## Data Availability Statement

The raw data supporting the conclusions of this article will be made available by the authors, without undue reservation.

## Ethics Statement

The studies involving human participants were reviewed and approved by the Ethics Committee of Hunan Cancer Hospital. Written informed consent for participation was not required for this study in accordance with the national legislation and the institutional requirements.

## Author Contributions

(I) Conception and design: BZ, HX, QX, WW; (II) Administrative support: BZ; (III) Provision of study materials or patients: BZ, JW, DY, JT, XL, ZW, YZ, WW; (IV) Collection and assembly of data: BZ, HX, QX; (V) Data analysis and interpretation: BZ, HX, QX; (VI) Manuscript writing: all authors; All authors contributed to the article and approved the submitted version.

## Funding

This study was supported in part by the Natural Science Foundation of Hunan Province (2019JJ40179); Scientific Research Project of Hunan Provincial Health and Family Planning Commission (20201743); and Changsha Science and Technology Project (kq1901079).

## Conflict of Interest

The authors declare that the research was conducted in the absence of any commercial or financial relationships that could be construed as a potential conflict of interest.

## Publisher’s Note

All claims expressed in this article are solely those of the authors and do not necessarily represent those of their affiliated organizations, or those of the publisher, the editors and the reviewers. Any product that may be evaluated in this article, or claim that may be made by its manufacturer, is not guaranteed or endorsed by the publisher.
